# Investigating the Role of DUSP4 in Uveal Melanoma

**DOI:** 10.1167/tvst.11.12.13

**Published:** 2022-12-28

**Authors:** Karen Aughton, Dorota Sabat-Pośpiech, Samantha Barlow, Sarah E. Coupland, Helen Kalirai

**Affiliations:** 1 Liverpool Ocular Oncology Research Group, University of Liverpool, Molecular and Clinical Cancer Medicine, Institute of Systems Molecular & Integrative Biology, University of Liverpool, Liverpool, UK; 2 Liverpool Clinical Laboratories, Liverpool University Hospital Foundation Trust, Liverpool, UK

**Keywords:** uveal melanoma, metastasis, DUSP4, immunohistochemistry, siRNA, doxorubicin, selumetinib, BAP1

## Abstract

**Purpose:**

Dual-specificity phosphatase 4 (DUSP4) inactivates factors in the mitogen-activated protein kinase (MAPK) signaling cascade, activated in uveal melanoma (UM) by mutations in upstream G-protein α subunits *GNAQ/11* in >90% cases. This study examined whether DUSP4 (1) protein expression in primary UM (pUM) was a biomarker of metastatic risk and (2) knockdown sensitized UM cells to therapeutic agents, selumetinib or doxorubicin.

**Methods:**

*DUSP4* mRNA data from The Cancer Genome Atlas and DUSP4 protein expression examined using immunohistochemistry in 28 cases of pUM were evaluated for association with clinical, genetic, and histological features. In vitro cytotoxic drug assays tested the efficacy of selumetinib and doxorubicin in UM cell lines with/without small interfering RNA *DUSP4* gene silencing.

**Results:**

DUSP4 protein expression was observed in 93% of cases, with strong nuclear positivity in 79%. Despite higher *DUSP4* messenger RNA levels in disomy 3/wild-type *BAP1* UM, there was no significant association of nDUSP4 protein with these metastatic risk predictors or outcome. DUSP4 expression in UM cell lines varied. *DUSP4* silencing in Mel202, MP46, and MP41 cells did not affect ERK1/2 or phospho-ERK levels. Despite increased phospho-ERK levels in Mel285, no cell line showed enhanced sensitivity to selumetinib/doxorubicin.

**Conclusions:**

DUSP4 protein expression is not a biomarker of UM metastatic risk. DUSP4 plays a complex role in oncogenesis, as reported in other cancers, and further work is required to fully understand its functional role in the MAPK pathway.

**Translational Relevance:**

Understanding the role of phosphatases, such as DUSP4, in the control of intracellular signaling cascades will facilitate our ability to identify successful treatment options.

## Introduction

Uveal melanoma (UM) is a rare primary adult intraocular tumor occurring in ∼6 to 8 individuals per million population annually.^1^ It has a high propensity to metastasize, usually to the liver, in approximately 50% of patients, resulting in high mortality.^2^ The median survival time is 6 to 12 months following metastatic onset,^2^ and there are limited effective treatment options available for metastatic UM (mUM).^3^

The genetic landscape of UM has a low mutational density compared with cutaneous melanoma,^4^ and more than 80% of UM have a driver mutation in the G-protein α subunit, *GNAQ* or *GNA11*,^5^ resulting in constitutive activation of downstream signaling pathways, including MAPK and PI3K/Akt.^6^^,^^7^ Additional, low-frequency mutations in two genes, *PLCB4* and *CYSLTR2*, leading to constitutively activated G-protein signaling, have also been reported in UM.^8^^,^^9^ Furthermore, inactivating mutations in BRCA1-associated protein 1 (*BAP1*) and somatic copy number variations (CNVs) (i.e., loss of one copy of chromosome 3 [monosomy 3] and gain of chromosome 8q) contribute to a high metastatic risk.^1^

Recent profiling of the transcriptomic landscape of mUM by our group, comparing 40 formalin-fixed paraffin-embedded mUM liver resections and 6 normal liver controls using NanoString technology, revealed 10 upregulated genes in mUM as compared with normal liver.^10^ The most highly differentially expressed gene was dual-specificity phosphatase 4 (*DUSP4*). Dual-specificity phosphatases (DUSPs) are a heterogeneous group of proteins that can be subdivided into six groups based on sequence homology, with DUSP4 belonging to the mitogen-activated protein kinase (MAPK) phosphatase group. DUSP4 dephosphorylates several proteins, including MAPK, and is involved in proliferation, differentiation, and apoptosis.^11^^–^^13^

DUSP4 has been reported as both downregulated and upregulated in many cancers^14^^–^^24^; therefore, implying its role in carcinogenesis is complicated. A role for DUSP4 in chemosensitization was demonstrated in both gastric^25^ and breast cancer,^26^ suggesting a mechanistic approach to overcome drug resistance.^27^ Other phosphatases reported to be upregulated in UM include protein tyrosine phosphatase 4A3 (PTP4A3), and high expression is predictive of poor outcome, highlighting the important role of phosphatases in UM.^28^^,^^29^

The aim of this study was to progress the findings of our previously published work^10^ by investigating the role of DUSP4 in UM, first as a potential biomarker for metastatic risk and second as a novel therapeutic target to enhance drug efficacy.

## Materials and Methods

This study conformed to the principles of the Declaration of Helsinki, and all procedures and methods were approved by both the Health Research Authority under the REC Ref 15/SC/0611 and the University of Liverpool Clinical Directorate sponsor Ref UoL001154. All samples and pseudo-anonymized data were provided by the Ocular Oncology Biobank (REC ref 21/NW/0139). All patients had provided informed consent for the use of their samples and data in research.

### Specimens

Formalin-fixed paraffin-embedded (FFPE) UM specimens (*n* = 28) were obtained from consented patients who had undergone primary enucleation for UM. Genetic characterization of these UM included CNV for chromosomes 3 and 8, as well as nuclear BAP1 (nBAP1) protein expression, for each case.

### Immunohistochemistry

FFPE blocks were sectioned at 4 µm and mounted on Superfrost microscope slides (ThermoFisher Scientific, Loughborough, UK). Slides were processed for immunohistochemistry using the Bond RXm Automated Stainer, incorporating antigen retrieval at pH 9.0, with the Bond polymer refine red detection system (Leica Biosystems UK Ltd, Milton Keynes, UK). DUSP4 antibody (ab72593; Abcam, Cambridge, UK) was used at a dilution of 1:100 (Table 1), with pancreas as the positive control.^10^

**Table 1.** Antibodies Used in the Study

Antibody	Antigen	Dilution	Species	Technique
Abcam Ab72593	DUSP4	1:100	Rabbit	IHC
Cell Signalling 5149S	DUSP4	1:500	Rabbit	WB
Invitrogen PA1-027A	Cyclophilin B	1:1000	Rabbit	WB
Cell Signalling 9102	ERK1/2	1:1000	Rabbit	WB
Cell Signalling 4370S	Phospho-ERK	1:1000	Rabbit	WB

IHC, immunohistochemistry; WB, Western blot.

### UM Cell Lines

The UM cell lines used in this study include 92.1, MP41, MP46, Mel202, and Mel285. Details of the five cell lines used are provided in Supplementary Table S1.^30^^,^^31^ All cells were maintained in RPMI 1640 with GlutaMax (Gibco, ThermoFisher Scientific) supplemented with 10% fetal calf serum (Labtech International Ltd, East Sussex, UK). Cells were incubated at 37°C with 5% CO_2_ humidity. All lines were mycoplasma free and used within 20 passages post resuscitation.

### Immunoblotting

Protein lysates were generated for each cell line by lysing cell pellets in RIPA buffer with 1% (v/v) phosphatase inhibitor (Phosphatase Inhibitor Cocktail 3; Merck, Gillingham, Dorset, UK) and 10% (v/v) protease inhibitor (cOmplete, Mini Protease Inhibitor Cocktail; Merck). Protein concentrations were measured using the Pierce BCA protein assay (ThermoFisher Scientific) according to the manufacturer's instructions. Western blots were run with 20 µg of protein per sample. Antibodies used are listed in Table 1 with cyclophilin B used as a loading control.^32^ Original membrane images shown in Supplementary Figures S1, S2, and S3.

### Drug Cytotoxicity Assay

Cells were plated in 96-well clear flat-bottomed plates at either 10,000 cells/well (92.1, MP41, Mel202) or 15,000 cells/well (MP46 and Mel285) for 24 hours. Doxorubicin was added at 0.5, 1, 5, and 10 µg/mL in 0.1% DMSO, with media and 0.1% DMSO control. Cells were incubated for 24-, 48-, and 72- hour time points before analysis. Selumetinib at a single maximal concentration of 30 µM in 0.1% DMSO was also tested in *DUSP4* small interfering RNA (siRNA) knockdown experiments.

### Sulforhodamine B Proliferation Assay

Cells were removed from the incubator and media discarded before fixing with 100 µL trichloroacetic acid (10%) at 4°C for 1 hour. Cells were then rinsed with water several times and allowed to air dry. Sulforhodamine B (SRB) solution (0.4% in 1% acetic acid) was added to the wells at 50 µL and allowed to stain cells for 1 hour at room temperature. Nonincorporated dye was washed with 1% acetic acid and the plate allowed to air dry. Incorporated dye was solubilized with 100 µL 10 mM Tris-Base and placed on a rocker for 10 minutes. Absorbance was measured using a spectrophotometer at a 565-nm wavelength with background absorbance measured at 690 nm.

### siRNA Transfection

For siRNA transfection optimization, Mel202, Mel285, MP46, and MP41 cell lines were plated into 6-well plates at a seeding density of 150,000 cells/well for 24 hours at 37°C. Cells were transfected with commercially available siRNA targeting DUSP4 (ON-TARGETplus DUSP4 siRNA smartpool; Dharmacon, Horizon Discovery, Cambridge, UK) at a final concentration of 20 nM (all cell lines) and 50 nM (MP46 and Mel285) with Optimem (Life Technologies, ThermoFisher Scientific) and Lipofectamine 2000 (Life Technologies) transfection reagents (knockdown [KD]). Standard off-target (OT) and RISC-free (RF) siRNAs (Dharmacon) were used as transfection controls in addition to a Lipofectamine only (LO) control. Cells were incubated for 24, 48, and 72 hours before harvesting using cold phosphate-buffered saline and cell pellets stored at −20°C ready for lysis for Western blotting. For subsequent doxorubicin/selumetinib experiments, the reagent volumes were adjusted for a 96-well plate format and final siRNA concentrations of 20 nM (Mel202 and MP41) and 50 nM (MP46 and Mel285) cell lines and incubated for 24 hours prior to drug additions for a further 24 hours.

### Statistics

Statistical analysis was performed using SPSS, version 27.0 (SPSS, Chicago, IL, USA) and GraphPad Prism (GraphPad Software, San Diego, CA, USA). Analysis included Student’s *t*-test, and survival curves were plotted using Kaplan–Meier methodology with status defined as death from metastatic melanoma. TCGA *DUSP4* messenger RNA (mRNA) data were analyzed using scatter boxplots comparing *DUSP4* mRNA expression with chromosome 3 status and the presence/absence of *BAP1* mutation; statistical significance was tested using an unpaired Student's *t*-test. For nuclear DUSP4 (nDUSP4) protein expression, receiver operating characteristic (ROC) curves were examined to inform any cutoff values used. The area under the curve values suggested poor sensitivity and specificity for model prediction, and due to the skewness of the data, the median value was used. Data for SRB cell cytotoxicity assays with doxorubicin/selumetinib and for cell proliferation were expressed as a percentage of the DMSO control with mean of three biological repeats represented for each experiment ± standard deviation. Data were analyzed using two-way analysis of variance with Bonferroni posttest comparing wild-type (WT) control, LO, OT, and RF with DUSP4 KD. Statistical significance was denoted as *P < 0.05, **P < 0.005, and ***P < 0.0005.

## Results

### DUSP4 Expression in Primary UM and Its Relationship With Clinicopathologic Features

**Figure 1.**
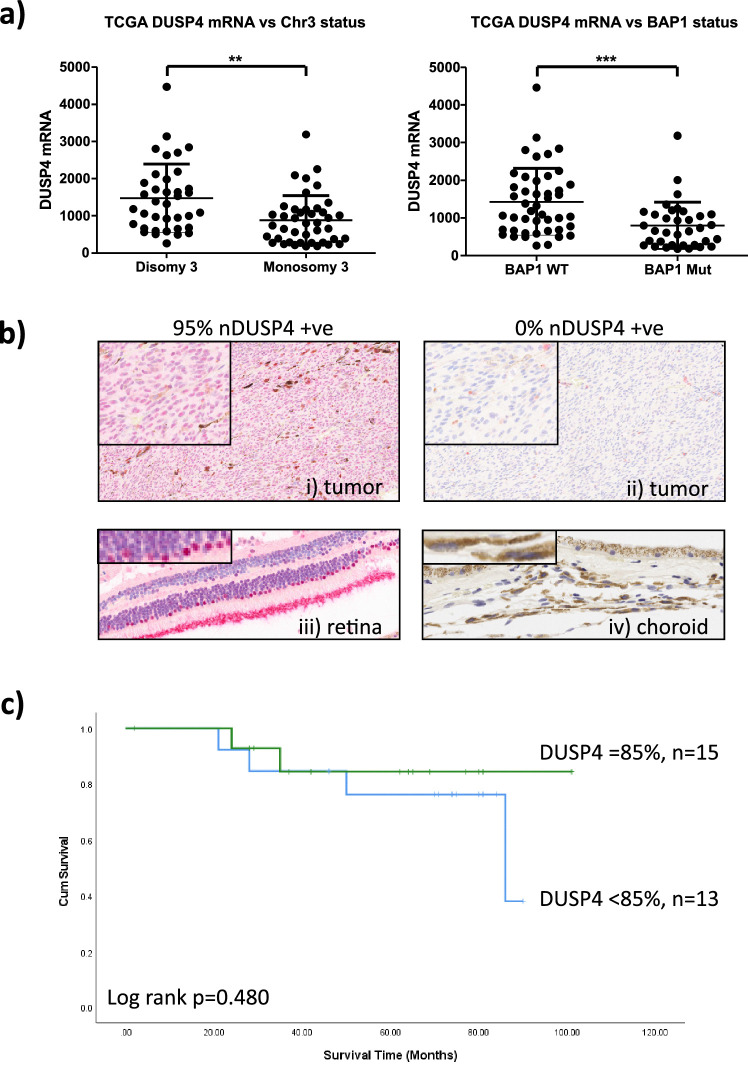
Expression of *DUSP4* mRNA and protein in primary UM samples with survival analysis. (a) TCGA mRNA scatter boxplot analysis showing UM *DUSP4* mRNA expression versus chromosome 3 status/*BAP1* (**P < 0.005, ***P < 0.0005). (b) DUSP4 pUM protein expression showing (i) strong nuclear staining and (ii) no nuclear staining, (iii) nDUSP4-positive retina as internal control, and (iv) nDUSP4-negative melanocytes (i, ii, iii: 20× magnification, iv: 40× magnification; all with a higher magnification thumbnail). (c) Kaplan–Meier survival plot showing no significant association with patient survival.

The Cancer Genome Atlas (TCGA) UM data demonstrate only 3 of 80 (∼4%) primary UM samples expressing high *DUSP4* mRNA. These data (downloaded from cBioPortal for Cancer Genomics^33^) are RSEM (RNA sequencing [RNA-Seq] by Expectation Maximization) normalized RNA-Seq data expressed as transcripts per million with a *z*-score threshold of 2 relative to UM samples analyzed in the cohort with a normal copy number for this gene.^5^ We undertook further quantile normalization of the RNA-Seq data using the 75th percentile and compared *DUSP4* mRNA expression with chromosome 3 status and the presence/absence of *BAP1* mutation. *DUSP4* mRNA levels were significantly higher in disomy 3 (*P* = 0.0019) and *BAP1* WT (*P* = 0.0004) UM (Fig. 1a).

To determine whether these data translated to protein expression, 28 patients with primary UM were chosen for this study based on nBAP1 protein immunohistochemical staining^34^: nBAP1 positive (*n* = 13) and nBAP1 negative (*n* = 15). As expected, monosomy 3 was significantly associated with nBAP1 protein loss (*P* = 0.007, data not shown).

DUSP4 protein expression was examined in the 28 samples by immunohistochemistry. Nuclear DUSP4 protein expression was seen in 26 of 28 (93%) cases (Fig. 1b). Cytoplasmic DUSP4 immunoreactivity was also seen in 12 of 28 UM samples, with weak to moderate expression being observed (data not shown). Nuclear DUSP4 positivity was observed in acinar cells and islets of Langerhans cells of the pancreas positive control, while the retina served as an internal positive control for UM tissue antigenicity with positive staining in the photoreceptors and the cell nuclei of the outer retinal layer (Fig. 1b). Normal choroidal melanocytes present in the tumor eyes were negative for nDUSP4 in 14 of 14 cases with areas of intact noninvolved choroid distant from the tumor (Fig. 1b).

ROC analyses failed to identify meaningful cutoff values according to area under the curve analyses. Due to the skewed nature of the data, the median nDUSP4 expression of 85% (range, 0%–95%) was used as the threshold (nDUSP4 <85%, *n* = 13; ≥85%, *n* = 15) to assess association with clinical, histological, and genetic features (Table 2). High nDUSP4 protein expression was significantly associated with older age (*P* = 0.02). A statistically significant association was not found between nDUSP4 expression and nBAP1 protein expression (*P* = 0.71), monosomy 3 (*P* = 1.0), or polysomy 8q (*P* = 0.085). Moreover, UM expressing nDUSP4 either above or below the median had no statistically significant association with patient survival (log rank = 0.48, Fig. 1c). Although these data suggest that nDUSP4 expression is not a predictive biomarker of metastasis or outcome, it may still play an important functional role in UM. Dysregulation of the phosphorylation-dephosphorylation cascade by DUSP4 expression could result in changes to downstream signaling or additional compensatory pathways leading to loss of efficacy of targeted agents.

**Table 2.** Clinicopathologic Features of Uveal Melanoma Samples With Low/High nDUSP4 Expression

Characteristic	Nuclear DUSP4 Low (<85%) (*n* = 13)	Nuclear DUSP4 High (≥85%) (*n* = 15)	*P* Value
Age (years)	62 (28–80)	68 (57–86)	**0.02**
Largest basal diameter (mm)	17.3 (12.0–21.7)	13.3 (9.0–22.0)	0.08
Tumor thickness (mm)	11.4 (6.5–16.3)	10.2 (6.6–14.5)	0.72
Cell type			
Epithelioid	10	10	0.69
Spindle	3	5	
PAS^+^ loops			
Presence	8	11	0.42
Absence	5	3	
Not known	0	1	
Mitotic count	4 (2–21)	5 (2–21)	0.39
Ciliary body involvement			
Yes	7	7	1.0
No	6	8	
BAP1			
Positive	7	6	0.71
Negative	6	9	
Chromosome 3			
Normal	3	3	1.0
Loss	10	12	
Chromosome 8q			
Normal	5	2	0.085
Gain	5	12	
Not known	3	1	

Age, largest basal diameter, tumor thickness, and mitotic count expressed as median (range) and analyzed using Student's *t*-test. All other analyses use Fisher's exact test. Significant values in bold. PAS, Periodic Acid-Schiff.

### DUSP4 Protein Expression and Sensitivity to Chemotherapeutic Agents in UM Cell Lines

To begin to address the role of DUSP4 in response to therapy, we examined DUSP4 expression in UM cell lines and their sensitivity to the MEK inhibitor selumetinib and the topoisomerase 2 inhibitor, doxorubicin. Previous studies by our group have shown selumetinib to have limited efficacy in UM cell lines^35^ despite targeting the constitutively activated MAPK pathway, a substrate protein for DUSP4. Additionally, doxorubicin has been reported in both breast^26^ and gastric^25^ cancer cell lines to show improved efficacy when DUSP4 expression is silenced. Therefore, we sought to discern the role of DUSP4 in the efficacy of these two compounds.

**Figure 2.**
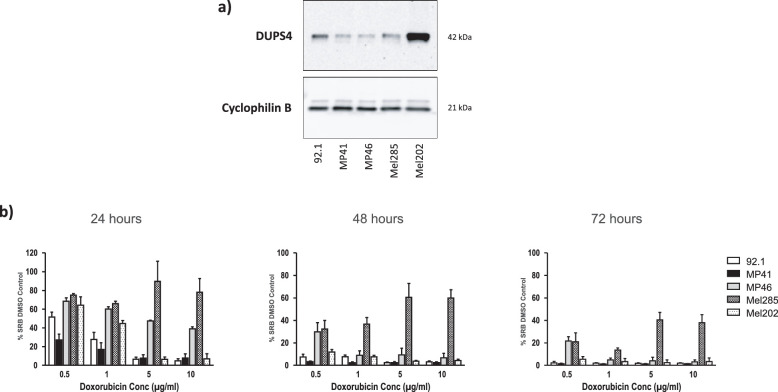
Expression of DUSP4 in UM cell lines and doxorubicin proliferation assays. (a) DUSP4 protein expression in UM cell lines (original blots are presented in Supplementary Figure S1) and (b) SRB cell proliferation assay in UM cell lines with doxorubicin over 24, 48, and 72 hours (mean ± SD, *n* = 3).

To examine the relationship between DUSP4 expression and doxorubicin sensitivity, a panel of UM cell lines was chosen for their genetic diversity, as seen in UM patients. DUSP4 protein expression was observed in 92.1, MP41, MP46, Mel202, and Mel285 cell lines using Western blot analysis (Fig. 2a), with Mel202 having the greatest DUSP4 expression (Mel202 > 92.1 > Mel285 > MP41 > MP46).

UM cell line sensitivity to doxorubicin was tested with dose-dependent increases in doxorubicin exposure for 24-, 48-, and 72- hour time points and cell number measured over time by SRB colorimetric end-point assay. Sensitivity to doxorubicin did not directly correlate with DUSP4 protein expression levels, and at 24 hours, MP46 and Mel285 showed the greatest resistance to doxorubicin with cell number reduced by 21.8% ± 14.4% and 61.1% ± 2.2%, respectively, at the highest concentration tested, 10 µg/mL (Fig. 2b). MP46 retained resistance at 72 hours to the lowest doxorubicin concentration of 0.5 µg/mL, and Mel285 showed the most prolonged resistance to doxorubicin at all concentrations such that at 72 hours, cell number was reduced by 62.0% ± 7.2% at 10 µg/mL doxorubicin. Mel202, 92.1, and MP41 showed the greatest sensitivity to doxorubicin with cell number reduced by 93.5% ± 2.4%, 93.3% ± 2.1%, and 92.3% ± 3.6%, respectively, at a lower concentration of 5 µg/mL.

### Sensitivity of UM Cell Lines to Doxorubicin or Selumetinib Is Unaffected by *DUSP4* Silencing

**Figure 3.**
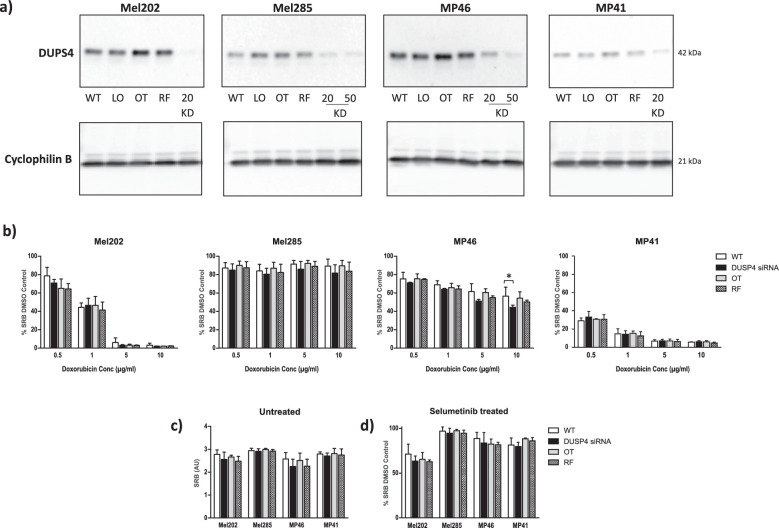
Effect of DUSP4 knockdown on doxorubicin and selumetinib sensitivity in UM cell lines. (a) *DUSP4* siRNA optimization in Mel202, Mel285, MP46, and MP41 UM cell lines at 24 hours (original blots are presented in Supplementary Figure S2). (b) SRB cell proliferation assay in UM cell lines ± *DUSP4* siRNA with doxorubicin. (c) Cell proliferation of untreated Mel202, Mel285, MP46, and MP41 UM cell lines ± DUSP4 knockdown (*P < 0.05). (d) SRB cell proliferation assay in UM cell lines ± *DUSP4* siRNA with selumetinib. (All mean ± SD, *n* = 3.) Controls—WT, LO, OT, and RF; knockdown at 20/50 nM (KD).

**Figure 4.**
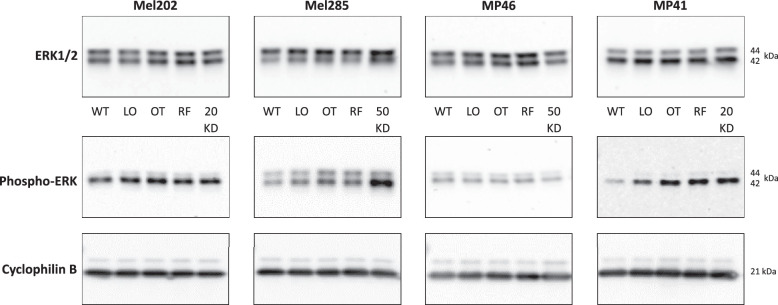
Effect of DUSP4 knockdown on ERK and phospho-ERK signaling in UM cell lines. Western blot analysis of ERK1/2 and phospho-ERK in Mel202, Mel285, MP46, and MP41 UM cell lines, including cyclophilin B loading control (original blots are presented in Supplementary Figure S3). Controls—WT, LO, OT, and RF; knockdown at 20/50 nM (KD).

For analysis of the effect of *DUSP4* silencing on doxorubicin sensitivity, UM cell lines with constitutive activation of signaling through G-protein coupled receptors due to mutations in *GNAQ/11* coupled with high metastatic risk features were used (i.e., Mel202 [*GNAQ* and *SF3B1* mutation], MP46 [*GNAQ* and *BAP1* loss], MP41 [*GNA11* mutation]) and compared with the *GNAQ/11* wild-type UM cell line Mel285. The siRNA concentration and incubation time was optimized for each cell line (Fig. 3a); 20 nM siRNA for Mel202 and MP41 and 50 nM for MP46 and Mel285 for 24 hours were chosen and drugs added at this time point for a further 24 hours. *DUSP4* remained silenced during the experimental period of 48 hours (data not shown). DUSP4 knockdown had no or minimal effect on doxorubicin sensitivity for any cell line or concentration compared with controls (Fig. 3b). The effect of DUSP4 knockdown on proliferation after 48 hours was also examined and demonstrated that there was no significant change to proliferation rates of knockdown cells compared with controls (Fig. 3c). DUSP4 is responsible for the dephosphorylation and inactivation of MAPK family members, including ERK. Thus, the role of DUSP4 activity on the MAPK pathway was assessed using Western blot analysis of DUSP4 knockdown in Mel202, MP46, Mel285, and MP41 UM cell lines probed with ERK1/2 and phospho-ERK antibodies. DUSP4 knockdown showed no change in expression of ERK1/2 (Fig. 4) when compared with controls in each of the cell lines. Phospho-ERK expression was unchanged between DUSP4 knockdown and OT and RF controls in Mel202, MP46, and MP41 cell lines (Fig. 4). This is consistent with the observation that DUSP4 knockdown did not affect sensitivity to selumetinib (Fig. 3d). Interestingly, in the *GNAQ/11* WT cell line, Mel285, DUSP4 knockdown increased the expression of phospho-ERK. However, similar to the other three cell lines, no increase in sensitivity following DUSP4 knockdown was observed (Fig. 3d).

## Discussion

To our knowledge, this is the first study that has examined the role of DUSP4 in UM in detail. Our analysis of the TCGA data demonstrated that *DUSP4* mRNA levels are higher in *BAP1* WT/disomy 3 primary UM. Our previous data also showed high nDUSP4 protein expression in 18 of 19 hepatic mUM samples,^10^ suggesting a correlation between this factor and metastasis. DUSP4 protein expression was thus examined in primary UM (pUM) of known nBAP1 protein status. Our main findings are that nDUSP4 protein expression was present in pUM but was not observed in normal choroidal melanocytes; in pUM, 93% of cases presented with nDUSP4 expression, with strong nuclear positivity (defined by presence in >50% of tumor cells) in 79%. However, no significant association of nDUSP4 protein expression with predictors of metastatic risk, including nBAP1 protein expression, monosomy 3, or outcome, was found in this current study. This suggests that *DUSP4* mRNA levels are not directly correlated with protein expression possibly due to mRNA/protein turnover and/or protein half-life. Despite an absence of correlation with metastatic risk in UM, DUSP4 plays a complex role in oncogenesis as previously reported in other cancers, acting as both a proto-oncogene or tumor suppressor,^14^^,^^36^^,^^37^ with effects on cell survival and proliferation depending on tissue and molecular subtype.^27^ This study also ascertained that DUSP4 did not enhance the efficacy of therapeutic agents tested here, again in contrast to previously reported studies, which further highlights the complexities of the role of phosphatases in UM.

DUSP4 is responsible for the dephosphorylation and inactivation of MAPK family members, in particular ERK1/2, p38, and JNK.^12^^,^^13^^,^^38^ In UM, there is a high frequency (>90% cases) of GNAQ and GNA11 mutations,^39^ leading to enhanced MEK-ERK1/2 signaling.^40^^,^^41^ Despite this, MEK inhibitors have had limited clinical efficacy in UM patients.^40^^,^^42^^,^^43^ Additionally, a study in zebrafish suggested that there was a weak correlation with *GNAQQ209P* mutation and ERK1/2-MAPK sustained activation,^44^ highlighting that MAPK may not be the dominant contributing pathway to continual cell proliferation. Moreover, upregulation of DUSP4 may suggest a continual cycling of aberrant phosphorylation-dephosphorylation events that impair the effectiveness of targeted agents and/or result in the activation of additional downstream or parallel compensatory signaling pathways.

To begin to address the role of DUSP4 in response to therapy, we examined DUSP4 expression in UM cell lines and their sensitivity to the MEK inhibitor selumetinib and the topoisomerase 2 inhibitor, doxorubicin. DUSP4 expression was observed in all UM cell lines examined, being highest in Mel202. No correlation was observed between DUSP4 protein levels and the sensitivity of the UM cell lines to either doxorubicin or selumetinib. In *BRAF* WT skin melanoma, patients expressing high levels of *DUSP4* mRNA had a better response to selumetinib.^45^ Subsequent investigation in *BRAF* WT melanoma cell lines indicated that *DUSP4* depletion enhanced cell survival and decreased sensitivity to MEK inhibition.^45^ In contrast, studies in breast and gastric cancer demonstrated that siRNA-mediated depletion of DUSP4 resulted in sensitization of cell lines to doxorubicin.^25^^,^^26^ Our data demonstrate that DUSP4 knockdown in UM cells with poor prognosis biomarkers and differing *GNAQ* mutation status (Mel202 *SF3B1* mutant, *GNAQ* mutant; MP46 *BAP1* mutant, *GNAQ* mutant; Mel285 *GNAQ* WT; MP41 *GNA11* mutant) had no effect on sensitivity to either therapeutic agent. Proliferation was similarly unaffected by DUSP4 knockdown, which contrasts with what has been observed in other cancer types, including colorectal cancer.^14^^,^^18^

Previous studies have shown selumetinib to have an effect on phospho-ERK expression with decreases seen in cell-based assays and PDX (patient-derived xenograft) tumor models of *GNAQ/11* mutant UM.^41^^,^^46^ In this study, we observed no changes in phospho- or total ERK expression in DUSP4 knockdown compared with controls in our *GNAQ/11* mutant cell lines. However, the increase in phospho-ERK observed in the Mel285 *GNAQ/11* wild-type UM cell line following DUSP4 knockdown indicates differences in the homeostasis of the downstream signaling components of the MAPK pathway in *GNAQ/11* mutant versus WT cells. The lack of effectiveness of DUSP4 knockdown to alter sensitivity to selumetinib in any of the cell lines highlights the probability that multiple phosphatases are involved. Indeed, a recent finding of dual *DUSP4/6* inactivation in *NRAS* and *BRAF* mutant cells supports this idea of compensatory gene relationships in the MAPK pathway.^47^

In conclusion, DUSP4 protein expression is upregulated in UM regardless of mutational and chromosomal aberrations. This study further reinforces the complexities of the role of DUSP4 in the MAPK signaling pathway and highlights potential tumor-intrinsic adaptive mechanisms for the control of intracellular signaling cascades by phosphatases.^48^ Further investigation of this will enable more informed approaches to optimize therapeutic strategies for mUM.

## Supplementary Material

Supplement 1

Supplement 2

Supplement 3

Supplement 4
