# Supplementary material for: Investigating the Role of DUSP4 in Uveal Melanoma
Source: Transl Vis Sci Technol. 2022 Dec 28;11(12):13. doi: 10.1167/tvst.11.12.13 (PMC9804032; doi:10.1167/tvst.11.12.13)
Supplement: Supplement 1 [file tvst-11-12-13_s001.pdf]

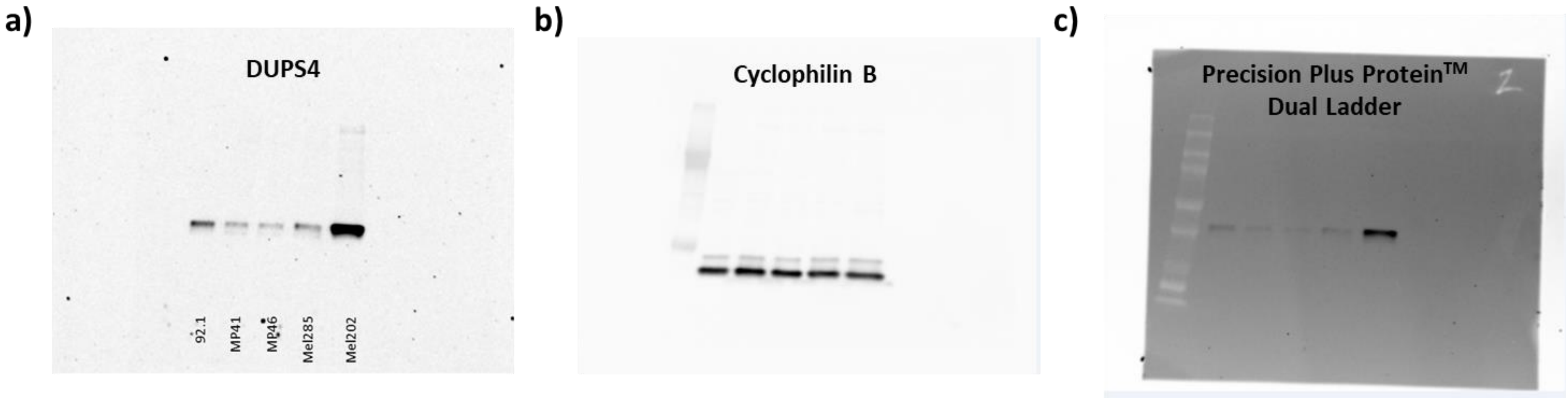

Supplementary Figure 1. Western blots - full membrane.

a) Cell line panel probed with DUSP4; b) Cell line panel probed with cyclophilin B; c) Precision Plus Protein Dual Color Standards image. All membranes imaged using GeneGnome by Syngene.
