# Supplementary material for: Investigating the Role of DUSP4 in Uveal Melanoma
Source: Transl Vis Sci Technol. 2022 Dec 28;11(12):13. doi: 10.1167/tvst.11.12.13 (PMC9804032; doi:10.1167/tvst.11.12.13)
Supplement: Supplement 2 [file tvst-11-12-13_s002.pdf]

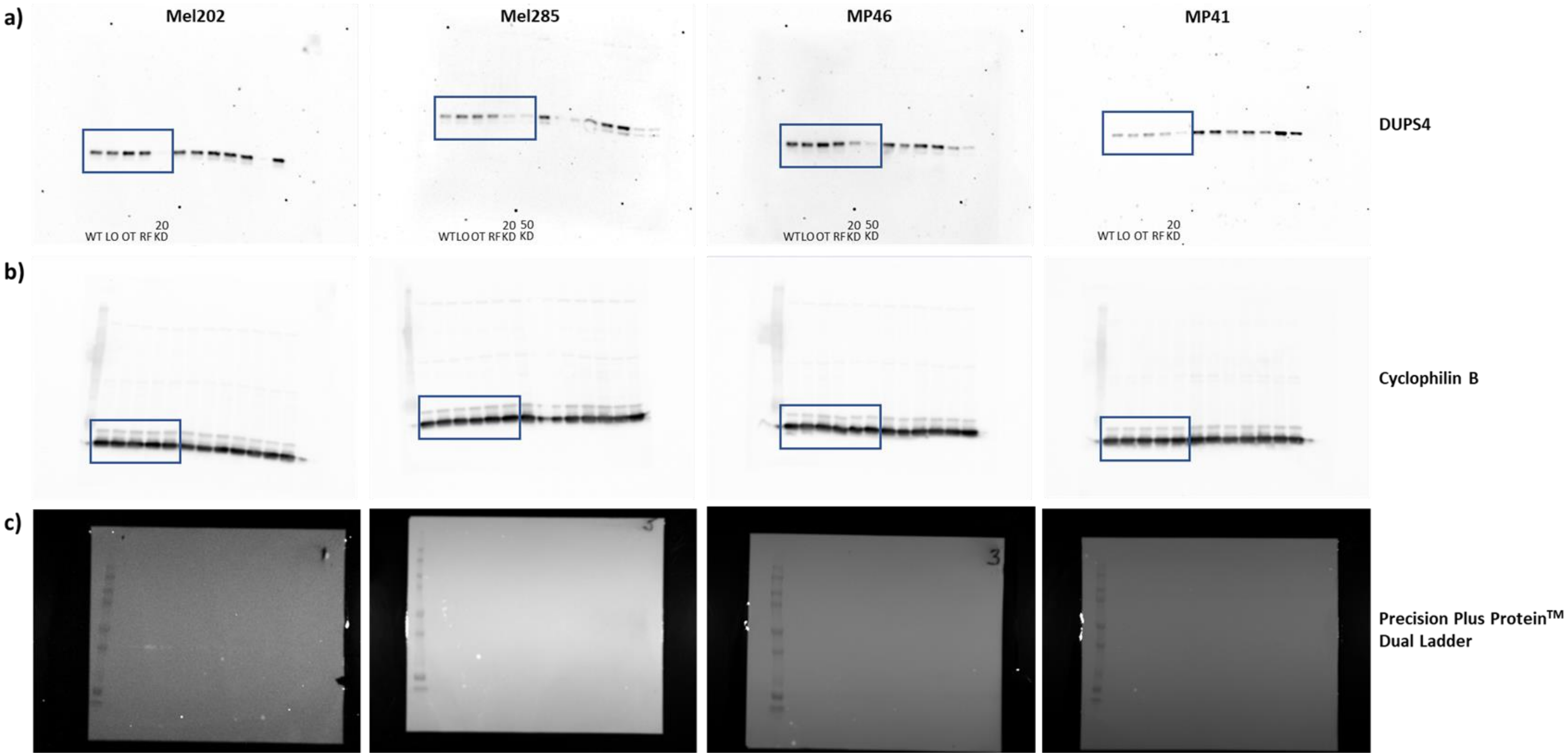

Supplementary Figure 2. Western blots - full membrane.

a) Cell knockdown membranes (Mel202, Mel285, MP46, MP41) probed with DUSP4; b) Cell knockdown membranes (Mel202, Mel285, MP46, MP41) probed with cyclophilin B; c) Precision Plus Protein^{TM} Dual Color Standards image. All membranes imaged using GeneGnome by Syngene.

Highlighted area on each membrane relates to data included in manuscript.

Controls - wild type (WT), lipofectamine only (LO), off target (OT), RISC free (RF), knockdown at 20/50 nM (KD).
