# Supplementary material for: Investigating the Role of DUSP4 in Uveal Melanoma
Source: Transl Vis Sci Technol. 2022 Dec 28;11(12):13. doi: 10.1167/tvst.11.12.13 (PMC9804032; doi:10.1167/tvst.11.12.13)
Supplement: Supplement 4 [file tvst-11-12-13_s004.pdf]

Supplementary Table 1: UM cell line characteristics

Cellular characteristics	UM Cell Line				
	92.1	MP41	MP46	Mel285	Mel202
**Origin**	Primary tumor	PDX established from primary tumor	PDX established from primary tumor	Primary tumor	Primary tumor
**Morphology**	Mixed	Mixed	Mixed	Mixed	Mixed
**nBAP1 (IHC)**	+	+	-	+	+
**Chr 3**	Partial losses	Uniparental disomy	Loss	Disomy	Disomy
**Chr 8q**	Gain	Gain	Gain	Gain	Gain
***GNAQ* mutation**	p.Q209L	WT	p.Q209L	WT	p.Q209L, p.R210K
***GNA11* mutation**	WT	p.Q209L	WT	WT	WT
***BAP1* mutation**	WT	WT	WT	WT	WT
***SF3B1* mutation**	WT	WT	WT	WT	p.R625G
***EIF1AX* mutation**	p.G6D	WT	WT	WT	WT

WT = wildtype; PDX = patient derived xenograft. Information included in the table is derived from in house analyses and/or the referenced sources.
